# Commercial complementary food feeding and associated factors among mothers of children aged 6–23 months old in Mettu Town, Southwest Ethiopia, 2022

**DOI:** 10.1186/s40795-023-00775-0

**Published:** 2023-10-24

**Authors:** Tirunesh Debessa, Zewudu Befkadu, Tefera Darge, Abeza Mitiku, Ebisa Negera

**Affiliations:** 1 Tullube Health Center, Mettu Woreda Health Office, Mettu, Ethiopia; 2 Department of Biomedical Sciences, College of Health Sciences, Mattu University, P.O. Box 318, Mettu, Ethiopia; 3 Department of Public Health, College of Health Sciences, Mattu University, Mettu, Ethiopia

**Keywords:** Commercial complementary food, Children aged 6–23 months, Mettu town

## Abstract

**Background:**

Commercial complementary foods (CCF) are unhealthy products for children under 24 months, containing unhealthy fats, refined starches, sugars, salt, and additives. The consumption of CCF is linked to non-communicable diseases, making it crucial to assess intake in Ethiopia, especially in Mettu town.

**Objective:**

To assess the prevalence of commercial complementary food feeding and associated factors among mothers of 6–23 months old children in Mettu Town, 2022.

**Method:**

A community-based cross-sectional study was conducted in Mettu town, involving 386 randomly selected mothers of children aged 6–23 months. Data was collected using a pre-tested semi-structured questionnaire and analyzed using SPSS version 25. Variables with a P-value < 0.05 in the multivariable logistic regression were declared as having a statistically significant association with CCF feeding.

**Results:**

The prevalence of CCF feeding within 24 h before the reporting period was 44.3%. In multivariable regression analysis, the age of index child 0-11months (AOR = 2.43, 95%CI: 1.53–3.85), non-exclusive breastfeeding (AOR = 2.18, 95%CI: 1.34–3.52), exposure to CCF promotions (AOR = 2.15, 95%CI: 1.32–3.50), maternal employment (AOR = 2.10, 95%CI: 1.28–3.44), and higher tertile wealth status (AOR = 2.19, 95%CI: 1.17–4.10) were significantly associated with CCF feeding.

**Conclusion:**

The current study revealed that nearly half of the mothers in Mettu town were feeding their children with commercially produced complementary foods. Age of child, non-exclusive breastfeeding, CCF promotions, maternal employment, and higher wealth status were found to have a significant association with CCF feeding. Therefore, continuous health education should be given to mothers to encourage exclusive breastfeeding until 6 months of age and to improve home-made complementary food feeding.

## Background

Commercial complementary foods (CCF) are any commercial food product, either wet (spoonable) or dry products like cereals and snacks, marketed for complementary feeding by children less than 24 months of age, as determined by the product label and directions of use [1]. These CCF are categorized as ultra-processed foods (UPF) according to the NOVA classification and are the main sources of highly unhealthy types of fat, refined starches, free sugars, salt, and additives [2].

Until six months old, an infant’s nutritional needs are met exclusively by breast milk; after this, the infant must start consuming additional foods and liquids [3]. Homemade infant foods containing fruits and vegetables (FV), and sources of vitamins, minerals, and fibers are recommended a complementary foods for infants and young children (IYC) [4]. Healthy eating habits are fundamental to the growth, development, and future health trajectories of young children. So, it is an important focus area for preventing future childhood malnutrition. Moreover, this period is a time when the infant’s eating habits and food preferences are more affected, which will continue into their adult life [5].

Alongside the growing global availability of processed foods and economic transition, consumption of CCF among IYC has been noted across developed and developing countries. Data from the European Childhood Obesity Project indicated that the proportion of children consuming any CCF was 68% at 24 months of age [6]. The prevalence of CCF feeding among children aged 6–23 months was also reported to be 24.9% in Brazil [7], 24.6% in Nepal [8], and 49% in Senegal [9].

In Ethiopia, commercially available complementary foods such as fafa, edget, cerifam, barley mix, famix, and favena are made using local cereals and legumes. These foods also include a vegetable protein concentrate and dried skimmed milk [10]. It is crucial to acknowledge that CCFs are fortified and contain the recommended levels of nutrients [11]. Nonetheless, it is still essential to remain cautious about potential health hazards. The incorporation of these processed foods into diets introduces foods typically high in sodium, sugar, and unhealthy fats that can potentially harm the quality of the diet and displace consumption of more nutrient-dense homemade foods [12–14].

The impact of CCF consumption on non-communicable diseases (NCDs), which is a global epidemic, is a relevant issue. In the past several decades, overweight and obesity have joined the longstanding high levels of stunting, forming a double burden of malnutrition (DBM) in sub-Saharan Africa [15, 16]. Studies have demonstrated a positive association between the consumption of UPFs and the risk of being overweight, obese, or having other related diseases [17].

Food contamination that may occur during manufacturing, packaging, transportation, and storage is another concern related to CCF. The results from a study done in China indicated mineral oil-saturated hydrocarbons (MOH) and polyolefin oligomer-saturated hydrocarbons were detected in most cereal-based foods, which is mostly due to their relatively higher fat contents. In addition to the ingredients, the food packaging represents another potential source of MOH in food [18].

In 2006, the Ethiopian Federal Ministry of Health released guidelines recommending the use of locally available recipes for preparing complementary foods for children aged 6–23 months [4]. These traditional Ethiopian foods include gruel, porridge, fetfet, kitta, and dabbo, which are made from grains like teff, sorghum, barley, maize, wheat, enset, and legumes like broad beans, chickpeas, field peas, and lentils [19]. Despite challenges such as food contamination during preparation and a lack of knowledge about creating balanced meals, these traditional foods are believed to provide the right amount of energy, balanced protein, essential amino acids, and necessary vitamins and minerals.

Like other developing countries, the transition from homemade complementary food to CCF feeding is a concern in Ethiopia, which needs to be studied to support it with evidence. Currently, there is no established national standard for CCF in the country. However, agro-food processing is the largest subsector, accounting for 36% of the total gross value of production and 33% of the national value-added of manufacturing industries in this country [20]. This creates opportunities for the introduction of unhealthy foods into the market system.

Considering the increasing participation of processed foods in the diet of children and the impacts associated with their consumption, assessing the intake of CCF becomes necessary. Currently, there is no published data on the prevalence of CCF feeding among mothers with under two-year-old children in Ethiopia, particularly in Mettu Town. Therefore, the aim of this study was to assess CCF feeding among mothers of 6- to 23-month-old children in Mettu, southwest Ethiopia, and to determine the factors that influence its consumption.

## Materials and methods

### Study setting and design

A community-based cross-sectional study was conducted in Mettu town, Ilubabour, southwest Ethiopia. It is located 600 km away from Addis Ababa, the capital city of Ethiopia. The town has been divided into six administrative kebeles with an estimated total population of 49,538 and 10,321 households. The estimated number of women in the reproductive age group was 9,214. Under-five and under-two age groups were estimated to be 8,139 and 2,828, respectively. The study was conducted from February 2 to April 21, 2022.

### Study population and sampling techniques

All mothers with index children aged 6–23 months old and living in Mettu town for at least six months were selected by a simple random sampling technique from the sampling frame. Mothers unable to respond due to illness were excluded.

The sample size was determined by using the single population proportion formula for a cross-sectional study, *n = Z*^*2*^*pq/d*^*2*^, considering a two-sided confidence level of 95%, a power of 80%, and a margin of error of 5%. The prevalence of CCF feeding among mothers of children aged 6–23 months old was taken as 50% because there is no published data from the previous study in the area. Accordingly, the calculated sample size was 384, and a total of 403 participants were selected by considering a 5% non-response rate.

**Fig. 1:**
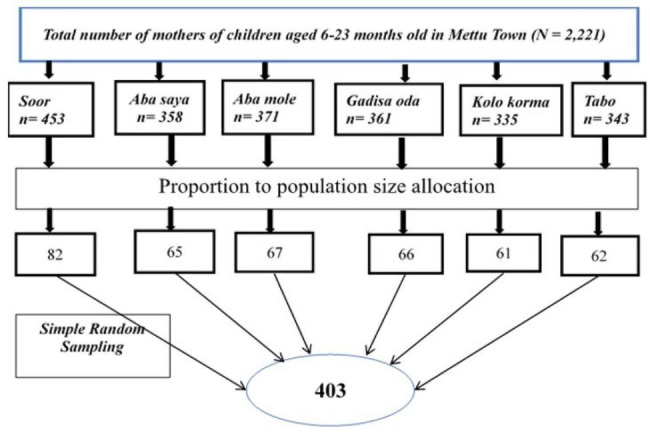
Schematic presentation of sampling procedure

Before data collection, a list of mothers of children aged 6–23 months was collected from each kebeles in Mettu town. Accordingly, a sampling frame was prepared, and the required number of participants from each kebeles was determined based on proportion to population size allocation. Finally, a simple random sampling technique was employed to select 403 study participants from the six kebeles (Fig. 1).

### Data collection tools and procedure

A structured, pretested, and interviewer-administered questionnaire was used to collect the data. The questionnaire was adapted from the World Health Organization (WHO), the Food and Agriculture Organization (FAO), and UNICEF guidelines and questionnaires [21–23]. The questionnaires consist of socio-demographic characteristics (age, sex, marital status, religion, educational status, wealth status, and occupation), maternal health service-related characteristics, child characteristics, early child feeding practices, and CCF practices.

The knowledge was measured using seven dichotomous questions aimed at gathering information regarding the impact of CCF feeding on a child’s overall health, its influence on their food preferences, the potential for contamination, and their understanding of its higher sugar content, added preservatives, and additives. The answer to each question was analyzed as know and don’t know. The mean score was calculated to report the knowledge level of the study participants. Participants who scored mean and above were categorized as having good knowledge, and those who scored less than the mean score were categorized as having poor knowledge.

To assess the attitude, participants were asked questions consisting of eight items centered around their views on whether CCF aids child growth, the convenience of using CCF, their contentment with feeding CCF to their child, and their endorsement of the notion that CCF enhances the child’s cognitive abilities. Each question was measured on a five-point Likert scale, which ranges from strongly agree to strongly disagree. Each scale was summated to evaluate the overall score, which represents the respondent’s position on the continuum of favorableness towards CCF feeding. Accordingly, 8 items have a potential minimum sum score of 8 to a maximum sum score of 40. When the total score of the participant is close to 40, it shows the most favorable attitude, and when the score is close to 8, it shows the most unfavorable attitude towards CCF feeding. Thus, based on this continuum of favorableness, it was categorized as negative if they scored between 8 and 23, neutral for those who scored 24, and positive for those who scored above 24.

Data were collected by six trained health extension workers and two experienced BSc Nurse supervisors who supervised the overall process of data collection. Data was collected by a house-to-house visit of mothers with an index child aged 6–23 months old. In the event that the eligible mother was absent from the home at the time of data collection, a revisit was done, and the mother who was absent at the third re-visit was considered a non-respondent.

### Operational definitions

- Complementary feeding: is the process of starting additional foods and liquids along with breast milk.
- Commercial complementary foods: foods marketed as suitable for feeding young children if they met at least one of the following criteria: (1) Marketed for introduction at an age of below three years; (2) labeled with the words “baby,” “toddler,” “young child,” or synonym; or (3) in any other way were presented as being suitable for children under the age of three years [24].
- Commercial complementary food feeding: feeding the child any CCF products on the day before the interview.

### Data quality assurance

Data were collected using pre-tested and properly designed questionnaires. To assure data quality, the questionnaires were prepared in English and translated into Afan Oromo and Amharic, then translated back to English by another person to check for consistency. Pre-testing of the questionnaires was performed using 5% of the sample size on mothers with similar socio-demographic characteristics living in Hurumu town, which was not the actual data collection area. Based on the pre-test, modifications were made to the questionnaire. The training was given to data collectors and supervisors on the aim of the study, data collection tools, data collection techniques, approach to the interviews, and maintaining the privacy and confidentiality of the respondents. Every day after data collection, questionnaires were reviewed and checked for completeness by the supervisors and principal investigator, and the necessary feedback was given to the data collectors each morning.

### Data processing and analysis

All data were checked visually, coded, and entered into Epi-data version 4.6 and exported to SPSS version 25 software packages for analysis. Descriptive statistics were calculated for variables. The results were presented in the form of tables, charts, and text using frequencies and summary statistics such as mean, standard deviation, and percentage to describe the study population with relevant variables. The degree of association between dependent and independent variables was assessed using an odds ratio with a 95% confidence interval. A simple binary logistic regression analysis was performed to select candidate variables for multivariable analysis. Variables with a P-value < 0.25 were taken as a cut-off point to select eligible variables for the multivariable logistic regression analysis, and variables with a p-value < 0.05 were declared as statistically significant in the final model. Pseudo-regression was performed to check the multi-collinearity between independent variables. For the final multivariable logistic regression model, the adequacy of the model to predict the outcome variables was checked by the Hosmer-Lemeshow goodness-of-fit test.

## Results

### Socio-demographic characteristics of the study participants

A total of 386 mothers with index children aged 6–23 months participated in the study. The mean age of the respondents was 29.6 years (SD ± 6.2), with a minimum age of 18 and a maximum age of 42 years. The mean age of the children was 13.61 months (SD ± 4.33), and more than half of the children, 241(62.4%), belongs to the age category of 12 months and above. Most of mothrs, 353 (91.5%), were married; 238 (61.7%) of them were secondary or above educational level; 38.6% of them were housewives; and 116 (30.1%) of them were employed. In terms of the wealth status of the respondents, 82 (21.2%) and 138 (35.8%) of them were ranked in the higher and lower tertiles, respectively.

### Maternal health service utilization and early child feeding practices

Almost all 371 (96.1%) of the respondents attended ANC service during the pregnancy of their current baby, and 174 (46.9%) of them had four or more ANC visits. Regarding counseling on child feeding, only 132 (35.6%) of them reported that they were counseled on child feeding during their ANC visit. The majority, 312 (80.8%) of them, received postnatal care within 24 h of their delivery, and 175 (56.1%) of them received counseling on child feeding during post-natal care visits. Regarding the obstetric experience of the participants, 246 (63.7%) of them were multi-para.

All of the children were breastfed at any time and 241 (62.4%) of mothers initiated breastfeeding within 1 h of delivery. Three hundred and fifty-nine (93%) of children were currently breastfeeding. All of the children were on complementary feeding, and 178 (46.1%) of the mothers reported that they had started providing food other than breast milk before the age of six months (Table 1).

**Table 1:** Maternal health service utilization and early child feeding practices of the study participants in Mettu town, Southwest Ethiopia, 2022

Variables	Category	Frequency (%)
ANC follow up	Yes	371 (96.1)
	No	15 (3.9)
Number of ANC visits (n = 371)	4 and above	174 (46.9)
	Less than four	197 (53.1)
Counseling on child feeding during ANC (n = 371)	Counseled	132 (35.6)
	Not counseled	239 (64.4)
PNC service	Yes	312 (80.8)
	No	74 (19.2)
Counseling on child feeding during PNC (n = 312)	Counseled	173 (55.4)
	Not counseled	139 (44.6)
Parity	Primi para	140 (36.3)
	Multipara	246 (63.7)
Breastfeeding initiation time	Within 1 h	241 (62.4)
	After 1 h	145 (37.6)
CF initiation time	Before 6 months	178 (46.1)
	At 6 months and above	208 (53.9)

ANC- Anti-natal care; PNC- Postnatal care; CF- Complementary food

### Knowledge and attitude of mothers toward commercial complementary feeding

Regarding the knowledge of respondents on the CCFs, 176 (45.6%) of the participants were categorized as having good knowledge, while 210 (54.4%) of them were categorized as having poor knowledge. Regarding the attitude of the participants toward commercial complementary feeding, 100 (25.9%) of them had a negative attitude, 252 (65.3%) of them had a positive attitude, and 34 (8.8%) of them had a neutral attitude toward CCF feeding.

### Commercial complementary food feeding and related factors

Among the total study participants, 171 (44.3%) of them have fed their child CCF in the last 24 h of the reporting period. Among these, 80 (46.8%) of them fed cereal-based products, 58 (33.9%) of them fed vegetables and fruit-based products, 29 (17%) of them fed milk-based products, and 4 (2.3%) of them fed meat and egg products. Two hundred and sixty-seven (69.2%) of the study participants reported that they had fed their child CCF within the last week of the reporting period. Among these, 147 (55.1%) of them fed at least for 3 days, while 120 (44.9%) of them fed for less than three days per week.

**Fig. 2:**
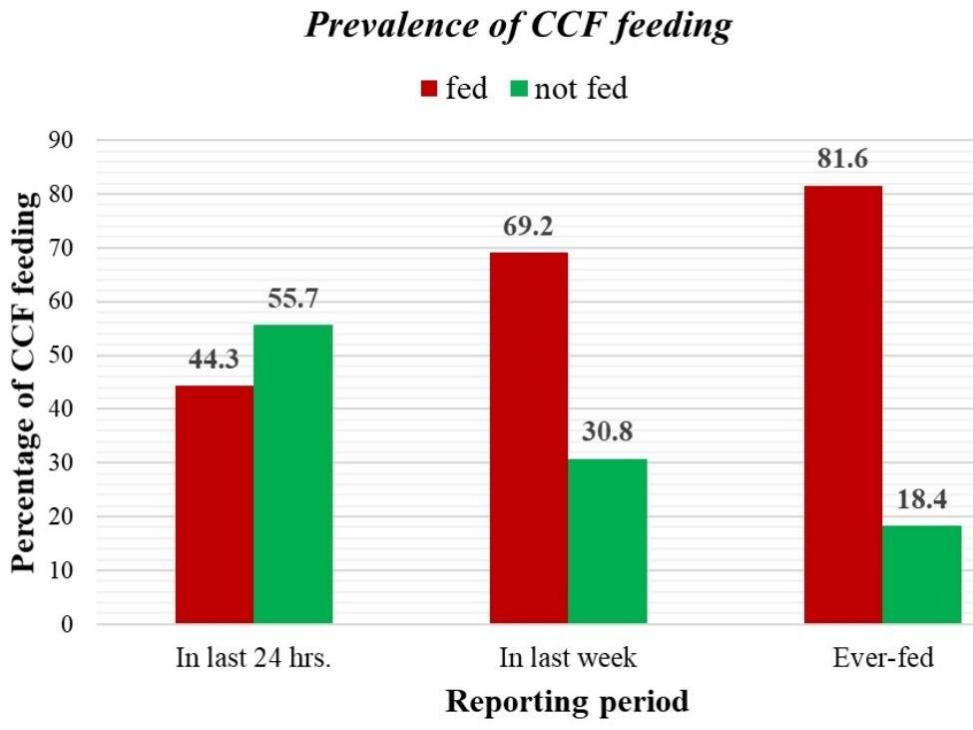
Prevalence of CCF feeding among mothers of children aged 6–23 months old, Mettu, southwest Ethiopia 2022

Besides, 315 (81.6%) of the respondents reported that they have ever fed their child with CCF. The average age for the initiation of CCF feeding was 5.89 months (SD ± 0.85), and 84(26.7%) of them started CCF feeding before the age of 6 months. The main reason for the initiation of CCF feeding was considering home-made complementary food as nutritionally insufficient (48.6%), followed by convenience (28.3%), less time to prepare homemade foods (18.4%), and other reasons such as weak or sluggish baby growth (4.7%) (Fig. 2).

### Factors associated with CCF feeding among the study participants

Binary logistic regression was employed to determine factors associated with CCF feeding. On bivariable analysis, maternal age, child age, child sex, non-exclusive breastfeeding, exposure to CCF promotions, parity, maternal attitude toward CCF, maternal occupation, and wealth status were significantly associated with CCF feeding within the last 24 h of the reporting period at *P*-value < 0.25. These nine independent variables were analyzed in multivariable logistic regression, and in the final model, child age, non-exclusive breastfeeding, exposure to CCF promotions, maternal occupation, and family wealth status were significantly associated with CCF feeding within the last 24 h of the reporting period at a *P*-value < 0.05.

Compared to those mothers with children aged 12–23 months old, mothers with children aged 0–11 months old were 2.43 times more likely to feed CCF (AOR = 2.43, 95% CI: 1.53–3.85). Mothers who didn’t exclusively breastfeed were 2.18 times more likely to feed CCF compared to those who exclusively breastfed (AOR = 2.18, 95% CI: 1.34–3.52). Compared to mothers who had no exposure to CCF promotions, those who were exposed to CCF promotions since the birth of their current baby were 2.15 times more likely to feed their child with CCF (AOR = 2.15, 95% CI: 1.32–3.50). Compared to unemployed mothers, employed mothers were 2.10 times more likely to feed their children with CCF (AOR = 2.10, 95% CI: 1.28–3.44). Compared to mothers from households with lower tertile wealth status, mothers from households with higher tertile wealth status were 2.19 times more likely to feed CCF (AOR = 2.19, 95%CI: 1.17–4.10) (Table 2).

**Table 2:** Factors associated with CCF feeding among mothers of children aged 6–23 months, Mettu, southwest Ethiopia 2022

Variable	Category	CCF feeding in 24 h.			
		Yes (%)	No (%)	COR (95% CI)	AOR (95% CI)
Age of mother (year)				1.03 (0.99–1.06)	0.98(0.93–1.03)
Child age	< 11 months	86(22.3)	59(15.3)	2.67 (1.75–4.08)	2.43 (1.53–3.85) *
	≥ 12 months	85(22)	156(40.4)	1	1
Child sex	Male	72(18.7)	106(27.5)	1	1
	Female	99(25.6)	109(28.2)	1.33 (0.89–2.00)	1.27 (0.80–2.03)
Not-exclusively breastfed	No	68(17.6)	140(36.3)	1	1
	Yes	103(26.7)	75(19.4)	2.82 (1.86–4.28)	2.18 (1.34–3.52) *
CCF promotions	No	56(14.5)	140(36.3)	1	1
	Yes	115(29.8)	75(19.4)	3.83 (2.5–5.86)	2.15 (1.32–3.50) *
Parity	Primi para	54(14)	86(22.3)	1	1
	Multipara	117(30.3)	129(33.4)	1.44 (0.94–2.20)	1.14 (0.70–1.85)
Maternal attitude	Negative	38(9.8)	62(16)	1	1
	Neutral	15(3.9)	19(4.9)	1.28 (0.58–2.83)	1.14 (0.46–2.78)
	Positive	118(30.6)	134(34.7)	1.43 (0.86–2.30)	0.85 (0.48–1.50)
Maternal occupation	Unemployed	102(26.4)	168(43.5)	1	1
	Employed	69(17.9)	47(12.2)	2.41 (1.55–3.77)	2.10 (1.28–3.44) *
Wealth status	Lower tertile	52(13.5)	86(22.3)	1	1
	Middle tertile	71(18.4)	95(24.6)	1.23 (0.78–1.96)	1.42 (0.84–2.39)
	Higher tertile	48(12.4)	34(8.8)	2.33 (1.33–4.08)	2.19 (1.17–4.10) *

* Statistically significant at p-value < 0.05

## Discussion

Consumption of commercially produced food products, often of limited nutritional value, carries implications for future child health and nutritional outcomes, including overweight/obesity and increased risk for non-communicable diseases. Hence, this study aimed to assess the prevalence of CCF feeding and associated factors among mothers of children aged 6–23 months old in Mettu town. Accordingly, the present study found that the prevalence of CCF feeding in the study area was 44.3%. Upon observing the figure, it appears to be significantly large. We speculate that the exclusion of rural areas and the focus solely on urban population might account for this outcome.

Our finding is comparable with the study conducted in Dakar, Senegal (49.1%) [9]. But it is lower than the multi-center study conducted in Europe (68%) [6] and a longitudinal study conducted in Germany (59.3%) [25]. The difference might be due to differences in socioeconomic status among study participants and study design. The cross-sectional study design is used in this study, while studies done in Europe and Germany used a longitudinal study design. On the other hand, the finding of the present study is greater than the findings of the cross-sectional studies conducted in Brazil (24.9%) [7] and Nepal (24.6%) [8]. The possible justification for the discrepancy might be differences in socioeconomic and demographic characteristics among study participants and study period.

It is interesting to note that of the mothers who fed CCF to their child, the majority (46.8%) of them fed their child cereal-based products. This finding aligns with the general perception that cereals are often recommended as a first food for infants due to their easy digestibility and nutrient content [26]. One potential negative consequence of commercially produced cereals for child feeding is the high sugar content often found in these products. Many cereals marketed for infants and young children contain added sugars, which can contribute to excessive calorie intake and increase the risk of developing conditions such as obesity and dental caries [27].

The age of the index child was found to be significantly associated with CCF feeding among the study participants. Mothers with index children below 11 months were two and a half times more likely to feed CCF compared to mothers with index children aged 12–23 months old. The possible explanation might be children under 11 months mostly feed liquid and semi-solid foods, which are the most common preparation forms of commercially produced food products. Besides, cereals are also frequently a baby’s first solid food introduction. This finding is similar to the study done in Nepal [8]. But it is contrary to a study done in Brazil [7], which showed that infants aged 6–11 months are less likely to consume CCF than children older than 12 months. The inconsistency could be due to the difference in socio-economic and demographic variables.

Providing foods other than breastmilk before age of 6 months is also associated with CCF feeding. Mothers who didn’t exclusively breastfeed were two times more likely to feed CCF when compared to those who exclusively breastfed. This finding is supported by studies done in Germany [25], Europe [6], and Brazil [28] which reported exclusive breastfeeding was associated with lower odds of consuming CCF. The possible reason could be as complementary feeding is initiated earlier, the chance of adapting commercially produced soft food products may increase. This trend will influence the family to continue providing commercially produced food for complementary feeding. In addition, early exposure to commercially manufactured milk provides opportunities for commercial food flavor learning and this might increase later product preference.

Mothers who had seen or heard of any CCF promotions had a two-fold increase in the likelihood of feeding CCF. Even though we couldn’t get any reports on the direct association of CCF promotion with the proportion of feeding, different studies showed that CCF is commonly promoted by using different media [9, 29]. The present study found that among the mothers who were exposed to CCF promotion since the delivery of their current child, 60% of them fed their child with CCF in the last 24 h of the reporting period. Food promotions focused on children are shown to correlate the products with fun and enjoyment, as well as a good flavor. This can influence the mothers’ perceptions of these products, including the belief that they are appropriate food for their young child, and negatively affect the appropriate and recommended child feeding practices.

The present study showed that the mother’s occupation is significantly associated with CCF feeding among the study participants. Mothers who were employed were two times more likely to feed their child CCF than unemployed mothers. This finding is supported by the CHOP study done in Europe [6] and the cross-sectional study done in Nepal [8]. This might be due to employed mothers being more scheduled with their outside work and spending most of their time outdoors. Due to this, they may not have adequate time to prepare homemade foods for their child.

Compared to those mothers from households with lower tertile wealth status, mothers from households with higher tertile wealth status were two times more likely to feed CCF. This finding is consistent with a study done in India [30]. This might be due to the fact that the preference for commercially produced food products can be influenced by the ability of the family to purchase them. The expensiveness of the products may limit mothers of lower tertile wealth status from feeding their children.

### Strength of the study

Using a community-based study design and being the first study on this topic in Ethiopia might be considered as strengths for the study.

### Limitation of the study

This study has its limitation. First, since the data were collected from the verbal reports of the participants, there might be a possibility of response bias. Second, the study didn’t assess the qualitative perspective of CCF feeding. Third, since it is a cross-sectional study, it couldn’t address the exact predictors of CCF feeding.

## Conclusion

The present study revealed that nearly half of the mothers with children aged 6–23 months in Mettu town were feeding their infants with commercially produced complementary foods. Age of index child (0–11 months), non-exclusive breastfeeding, exposure to CCF promotions, maternal employment, and higher tertile wealth status were found to have a significant association with CCF feeding. Hence, continuous health education should be given to mothers to encourage exclusive breastfeeding until 6 months of age and to improve home-made complementary food feeding. Longitudinal studies should be conducted to know the exact predictors of CCF feeding by considering the qualitative aspects.

## Data Availability

The datasets generated and/or analyzed during the current study are available from the corresponding author upon reasonable request.

## List of abbreviations

ANC: Anti-natal care
CCF: Commercial Complementary Foods
CF: Commercial Foods
CHOP: Childhood Obesity Project
DBM: Double Burden of Malnutrition
DONALD: Dortmund Nutritional and Anthropometric Longitudinally Designed
FAO: Food and Agriculture Organization
FV: Fruits and Vegetables
IYC: Infants and Young Children
LMIC: Low-Income and Middle‐Income Countries
MOH: Mineral Oil Saturated Hydrocarbons
NCDs: Non-Communicable Diseases
PNC: Post-natal care
UPF: Ultra-Processed Foods
WHO: World Health Organization

## References

[1] Maslin K, Venter C (2017). Nutritional aspects of commercially prepared infant foods in developed countries: a narrative review. Nutr Res Rev.

[2] Theurich MA, Humphreys AL, Gosselin LB, Mccool-Myers ME (2019). Food safety considerations for commercial complementary foods from global operational guidance on infant and young child feeding in emergencies. Nutr Rev.

[3] WHO. Infant and young child feeding [Internet]. 2021. p. 1–8.

[4] FDRE/MOH. Complementary Feeding Recipes for Ethiopian Children 6–23 Months Old A Practical Cooking and Feeding Guide. 2006;(August).

[5] D’auria E, Borsani B, Pendezza E, Bosetti A, Paradiso L, Zuccotti GV (2020). Complementary feeding: pitfalls for health outcomes. Int J Environ Res Public Health.

[6] Theurich MA, Zaragoza M, Luque V, Gruszfeld D, Gradowska K, Xhonneux A et al. Commercial complementary food use amongst European infants and children: results from the EU Childhood Obesity Project. Eur J Nutr. 2019;1–18. 10.1007/s00394-019-02023-3 31263982

[7] Marçal GDM, Moura M, Di M, Mafra G, Maria T, Toledo DM et al. Association between the consumption of ultra-processed foods and the practice of breast-feeding in children under 2 years of age who are beneficiaries of the conditional cash transfer programme, Bolsa Família. Public Health Nutr. 2020;1–9. 10.1017/S136898002000244X PMC10195259 32799961

[8] Pries AM, Huffman SL, Adhikary I, Upreti SR, Dhungel S (2016). High consumption of commercial food products among children less than 24months of age and product promotion in Kathmandu Valley, Nepal. Maternal and Child Nutrition.

[9] Feeley AB, Coly AN, Yaga N, Gueye S, Diop EI, Pries AM (2016). Promotion and consumption of commercially produced foods among children: situation analysis in an urban setting in Senegal. Matern Child Nutr.

[10] Melese T (2013). Nutritional Status of Ethiopian Weaning and Complementary foods: a review. Open Access Scientific Reports.

[11] Dewey K, Berger J, Chen J, Chen C, De Pee S, Huffman S (2009). Formulations for fortified complementary foods and supplements: review of successful products for improving the nutritional status of infants and young children. FoodNutr Bull.

[12] Baker P, Machado P, Santos T, Sievert K, Backholer K, Hadjikakou M (2020). Ultra-processed foods and the nutrition transition: global, regional and national trends, food systems transformations and political economy drivers. Obes Rev.

[13] Foterek K, Hilbig A, Alexy U (2015). Associations between commercial complementary food consumption and fruit and vegetable intake in children. Results of. Appetite.

[14] Foterek K, Buyken AE, Bolzenius K, Hilbig A, Nöthlings U, Alexy U (2016). Commercial complementary food consumption is prospectively associated with added sugar intake in childhood. Br J ofNutrition.

[15] Ng M, Fleming T, Robinson M, Thomson B, Graetz N, Margono C (2014). Global, regional, and national prevalence of overweight and obesity in children and adults during 1980–2013: a systematic analysis for the global burden of Disease Study 2013. The Lancet.

[16] Reardon T, Tschirley D, Liverpool-tasie LSO, Awokuse T, Fanzo J, Minten B (2021). The processed food revolution in African food systems and the double burden of Malnutrition. Global Food Security.

[17] Chen X, Zhang Z, Yang H, Qiu P, Wang H, Wang F (2020). Consumption of ultra-processed foods and health outcomes: a systematic review of epidemiological studies (Consumo De Alimentos Ultraprocessados ​​E resultados para a saúde: uma revisão sistemática de estudos epidemiológicos). Nutr J.

[18] Liu L, Li B, Yang D, Ouyang J, Sui H, Wu Y (2021). Food Additives & Contaminants: Part A Survey of mineral oil hydrocarbons in Chinese commercial complementary foods for infants and young children. Food Addit Contaminants: Part A.

[19] Federal Democratic Republic. Of Ethiopia. Food. 2014.

[20] Ahmed J, Tefera T, Kassie GT (2020). Consumers’ preference and willingness to pay for enriched snack product traits in Shashamane and Hawassa cities, Ethiopia. Agricultural and Food Economics.

[21] WHO and UNICEF (2021). Indicators for assessing infant and young child feeding practices. World Health Organization and the United Nations Children’s Fund (UNICEF).

[22] FAO. Guidelines for assessing nutrition-related K nowledge, a ttitudes and P ractices manual guidelines for assessing nutrition-related K nowledge, a ttitudes and P ractices manual [Internet]. Control. 2014. 1–188 p.

[23] World Health Organization. Essential Nutrition Actions: improving maternal, newborn, infant and young child health and nutrition. 2013;1–148. 25473713

[24] WHO, UNICEF. IBFAN. Marketing of breast-milk substitutes: National implementation of the international code. WHO. 2020.

[25] Foterek K, Hilbig A, Alexy U (2014). Breast-feeding and weaning practices in the DONALD study: age and time trends. J Pediatr Gastroenterol Nutr.

[26] Klerks M, Bernal MJ, Roman S, Bodenstab S, Gil A, Sanchez-Siles LM (2019). Infant cereals: current status, challenges, and future opportunities for whole grains. Nutrients.

[27] Vos MB, Kaar JL, Welsh JA, Van Horn LV, Feig DI, Anderson CAM (2017). Added sugars and Cardiovascular Disease risk in children: a scientific statement from the American Heart Association. Circulation.

[28] Spaniol AM, Helena T, Bortolini GA, Gubert MB (2020). Breastfeeding reduces ultra-processed foods and sweetened beverages consumption among children under two years old. BMC Public Health.

[29] Hadihardjono DN, Green M, Stormer A, Agustino, Izwardy D, Champeny M (2019). Promotions of breastmilk substitutes, commercial complementary foods and commercial snack products commonly fed to young children are frequently found in points-of-sale in Bandung City, Indonesia. Maternal and Child Nutrition.

[30] Bisoi SK, Mohanty MD, Dash DK, Giri S (2019). Complementary feeding practices and its economic and social impact: a cross sectional hospital based study. J Nepal Pediatr Soc.

